# Causal association between Parkinson’s disease and cancer: a bidirectional Mendelian randomization study

**DOI:** 10.3389/fnagi.2024.1432373

**Published:** 2024-11-05

**Authors:** Chunyan Tang, Ping Fu, Liangqing Lin, Hui Zhou, Yunjun Huang, Yang Li, Sijun Zhao

**Affiliations:** ^1^The Second Department of Neurology, Jiangxi Provincial People's Hospital, The First Affiliated Hospital of Nanchang Medical College, Nanchang, Jiangxi, China; ^2^Department of Traumatology, Jiangxi Provincial People's Hospital, The First Affiliated Hospital of Nanchang Medical College, Nanchang, Jiangxi, China

**Keywords:** Parkinson’s disease, cancer, Mendelian randomization, causal relationship, single-nucleotide polymorphisms

## Abstract

**Background:**

Previous observational research has indicated a correlation between Parkinson’s disease (PD) and multiple cancers; but the causality remains unclear. Thus, we utilized Mendelian randomization (MR) analysis to explore the potential causal link between PD and various cancers.

**Methods:**

We conducted a bidirectional two-sample Mendelian randomization (TSMR) of genetic variants associated with PD and 14 types of cancers. Summary statistics on PD and 14 types of cancers were obtained from the International Parkinson’s Disease Genomics Consortium and the study by Sakaue et al. The primary method employed was inverse variance weighted (IVW), complemented by multiple sensitivity analyses to evaluate heterogeneity and pleiotropy. The false discovery rate (FDR) was employed to control the false positive rate of multiple hypothesis testing.

**Results:**

Following rigorous sensitivity analyses and corrections, our findings revealed suggestive associations between PD and certain cancers. We observed that PD decreases the risk of gastric cancer and colorectal cancer (OR = 0.936, 95% CI = 0.881–0.995, *p* = 0.034, P FDR = 0.239; OR = 0.955, 95% CI = 0.912–0.999, *p* = 0.046, P FDR = 0.215), while increasing the risk of breast cancer (OR = 1.043, 95% CI = 1.004–1.084, *p* = 0.029, P FDR = 0.402). Notably, we found no evidence supporting a reverse causal relationship. Additionally, in the reverse pathway, skin cancer demonstrated a suggestive causal relationship with PD (OR = 0.913, 95% CI = 0.857–0.973, *p* = 0.005, P FDR = 0.066).

**Conclusion:**

Our MR analysis provides evidence supporting unidirectional suggestive causal relationships between PD and certain cancers. These findings enrich our comprehension of the intricate interplay between PD and cancer, warranting further investigation into the underlying biological mechanisms.

## 1 Introduction

Parkinson’s disease (PD) is a prevalent and intricate neurodegenerative condition, impacting approximately 1% of individuals aged 60 and above, with its prevalence expected to double within the next three decades (Antony et al., 2013; Tolosa et al., 2021). Its primary hallmark involves the degeneration of dopaminergic neurons in the substantia nigra pars compacta, leading to reduced dopamine levels in the brain and the formation of Lewy bodies. These processes precipitate typical motor symptoms such as tremors, bradykinesia, rigidity, and postural instability (Postuma et al., 2015; Jankovic and Tan, 2020; Leite Silva et al., 2023; Ye et al., 2023). Apart from these motor symptoms, PD often presents with various non-motor manifestations, including depression, cognitive impairment, sleep disturbances, and constipation (Schapira et al., 2017; Weintraub et al., 2022). Despite the significant burden PD places on families and healthcare systems worldwide, effective treatments remain elusive.

Cancer, similar to neurodegenerative disorders, presents as a chronic ailment. In various investigations, the prevalence of malignant tumors among individuals with PD appears to be lower compared to the general populace (Surguchov and Surguchev, 2024). A meta-analysis encompassing 50 observational studies uncovered a 17% reduction in the incidence of malignant tumors among PD patients, particularly notable in cancers such as lung, bladder, colorectal, prostate, endometrial, and hematologic malignancies (Catalá-López et al., 2014). Large cohort studies conducted in Taiwan have also indicated a negative association between PD and the majority of cancers (Lin et al., 2015).

Nevertheless, documented positive associations have been observed between PD and specific cancers, notably melanoma and brain cancer (Ryu et al., 2020; Sugier et al., 2023). A meta-analysis involving 40 sets of clinical studies revealed that individuals with PD had a reduced risk of lung, genitourinary, gastrointestinal, and hematological cancers. However, PD patients showed an elevated risk of melanoma and brain cancer (Leong et al., 2021). Another cohort study conducted in the United States suggested that, compared to the control group, PD patients had a 3.8-fold increased risk of developing melanoma; notably, individuals with melanoma also displayed a 4.2-fold higher risk of developing PD (Dalvin et al., 2017). Presently, observational studies may encounter bias due to inherent confounding factors, posing challenges in establishing causality. In this context, we undertook Mendelian randomization analysis (MR).

MR analysis is an effective approach for exploring causal relationships between two traits, presenting potential remedies for certain limitations of traditional observational studies. MR utilizes genetic variation as instrumental variables (IVs), effectively establishing a natural randomized controlled trial and enriching the arsenal of epidemiological research methodologies (Sekula et al., 2016). As genome-wide association studies (GWAS) continue to expand in scale and scope, the two-sample MR analysis has gained widespread acceptance across diverse disease contexts (Long et al., 2023; Valencia-Hernández et al., 2023; Dai et al., 2024; Shen et al., 2024; Yu et al., 2024). Consequently, we applied bidirectional TSMR analysis to evaluate the causal relationship between PD and multiple cancers, leveraging summary statistics derived from extensive GWAS datasets.

## 2 Materials and methods

**Figure 1:**
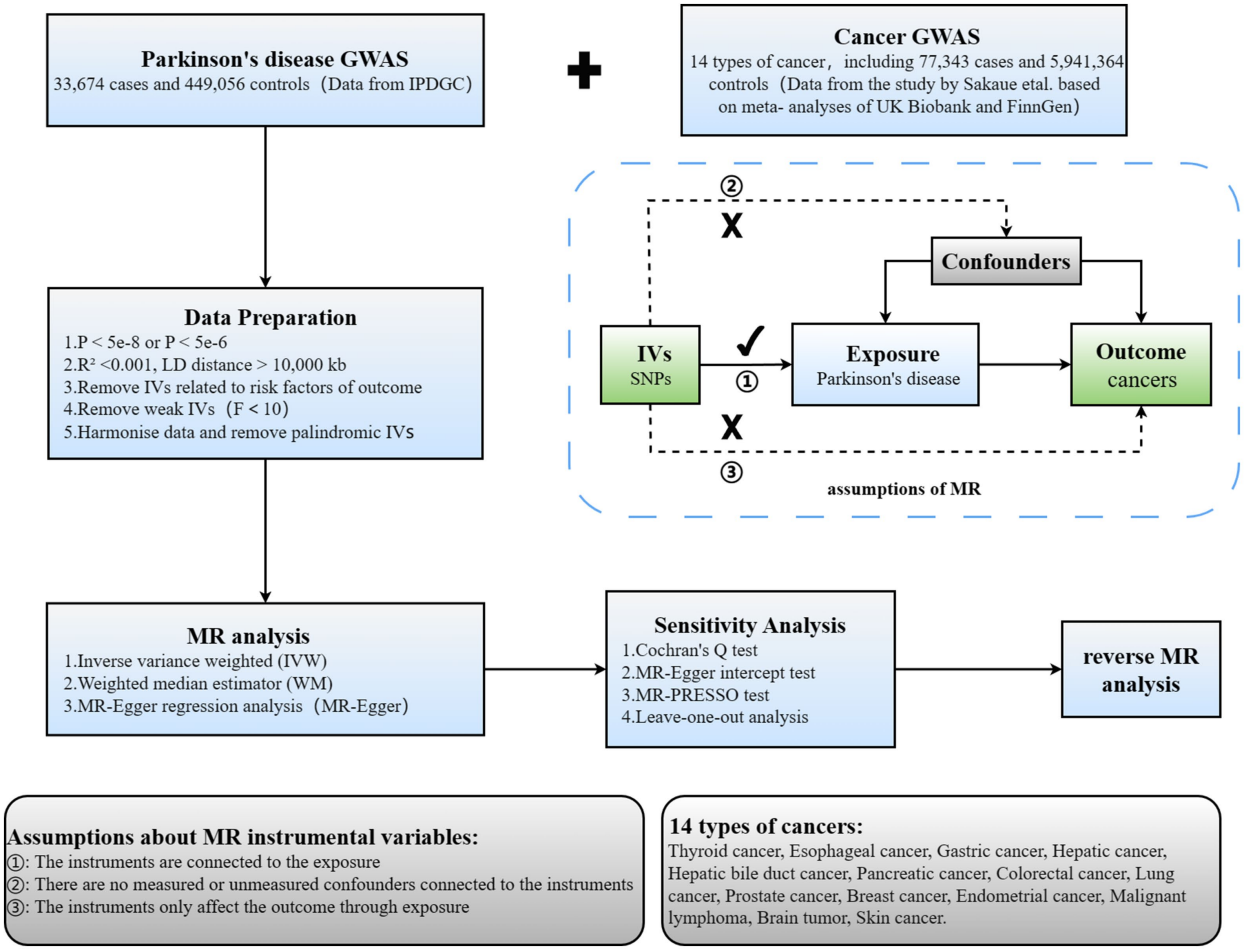
Overview of current MR analyses and assumptions. IPDGC, International Parkinson’s Disease Genomics Consortium; SNP, single-nucleotide polymorphism.

TSMR was employed to scrutinize the causal link between PD and 14 types of cancer. The comprehensive design of this investigation is illustrated in Figure 1. Three hypotheses must be met to ensure robust outcomes in TSMR analysis: (1) IVs must demonstrate a robust correlation with the exposure; (2) IVs should remain unassociated with any identified or unidentified confounding factors; (3) IVs should exclusively impact the outcome via the designated exposure and not through alternative pathways.

### 2.1 Summary data from GWAS on Parkinson’s disease and 14 types of cancer

To attain more dependable conclusions regarding causality, we accessed relevant data from the largest publicly available GWAS. Since all data used were have been previously published in public databases, ethical approval or informed consent was unnecessary. Specifically, we extracted statistical data on PD and 14 types of cancer from the IEU Open GWAS database (Table 1).

**Table 1:** Mendelian randomization study dataset details.

Variable	Case	Controls	Sample size	Year	Consortium	GWAS ID
Parkinson’s disease	33,674	449,056	482,730	2019	IPDGC	ieu-b-7
Thyroid cancer	1,054	490,920	491,974	2021	UKB + FG	ebi-a-GCST90018929
Esophageal cancer	998	475,308	476,306	2021	UKB + FG	ebi-a-GCST90018841
Gastric cancer	1,029	475,087	476,116	2021	UKB + FG	ebi-a-GCST90018849
Hepatic cancer	379	475,259	475,638	2021	UKB + FG	ebi-a-GCST90018858
Hepatic bile duct cancer	832	475,259	476,091	2021	UKB + FG	ebi-a-GCST90018803
Pancreatic cancer	1,196	475,049	476,245	2021	UKB + FG	ebi-a-GCST90018893
Colorectal cancer	6,581	463,421	470,002	2021	UKB + FG	ebi-a-GCST90018808
Lung cancer	3,791	489,012	492,803	2021	UKB + FG	ebi-a-GCST90018875
Prostate cancer	11,599	199,628	211,227	2021	UKB + FG	ebi-a-GCST90018905
Breast cancer	17,389	240,341	257,730	2021	UKB + FG	ebi-a-GCST90018799
Endometrial cancer	2,188	237,839	240,027	2021	UKB + FG	ebi-a-GCST90018838
Malignant lymphoma	3,546	487,257	490,803	2021	UKB + FG	ebi-a-GCST90018878
Brain tumor	833	490,709	491,542	2021	UKB + FG	ebi-a-GCST90018800
Skin cancer	25,928	466,275	492,203	2021	UKB + FG	ebi-a-GCST90018921

IPDGC, International Parkinson’s Disease Genomics Consortium; UKB + FG, UK Biobank and FinnGen Biobank.

Summary statistics for PD GWAS (33,674 patients with PD and 449,056 controls) were sourced from the International Parkinson’s Disease Genomics Consortium (IPDGC). Data for GWAS on 14 types of cancer (77,343 cancer patients and 5,941,364 controls) were sourced from a study conducted by Sakaue et al., based on meta-analyses of UK Biobank and FinnGen data (Sakaue et al., 2021). A bidirectional, TSMR study was proceed to explore the causal relationship between PD and cancer (Figure 1). Additionally, all study participants were of European descent, ensuring no overlap between the samples for exposure and outcome characteristics, thus minimizing bias attributable to confounding factors to the greatest extent possible.

### 2.2 Selection of instrumental variables

Based on the summary data from the previously mentioned GWAS, we rigorously adhered to a meticulous protocol to discern eligible single nucleotide polymorphisms (SNPs) to serve as IVs. Firstly, SNPs were required to demonstrate a robust association with the exposure, adhering to a genome-wide significance threshold established at *p* < 5e-8. Secondly, linkage disequilibrium (LD) clustering was proceeded to identify independent SNPs meeting the specified criteria (LD R^2^ < 0.001, LD distance = 10,000 kb). Thirdly, in reverse Mendelian randomization, to ensure an adequate number of shared SNPs between exposure and outcome, we relaxed the standard for instrumental variable selection, setting it at *p* < 5e-6 (Luo et al., 2022). Finally, the exposure and outcome datasets underwent harmonization to reduce the presence of symmetric and ambiguous SNPs, as well as SNPs with inconsistent alleles between exposure and outcome, thereby ensuring consistency of allele effects on exposure and outcome.

Moreover, to better fulfill key assumption one, the F-statistic was calculated for each SNP individually. Typically, weak instrumental variables are identified by F-statistics less than 10 and are thus excluded from further analysis. The R^2^ were computed using the following formulas:


$$R^{2}=2\times EAF\times \left(1-EAF\right)\times \beta^{2}$$

The F-statistic for each SNP was computed using the following formulas:


$$F=R^{2}\frac{\left(N-2\right)}{\left(1-R^{2}\right)}$$

*EAF*, *β*, and *N* correspond to effect allele frequency, effect size, and sample size, respectively (Burgess and Thompson, 2011). Subsequently, the carefully selected SNPs from the aforementioned screening steps were utilized as the final IVs for subsequent TSMR analyses.

### 2.3 Statistical analysis

The inverse variance weighted (IVW) method served as the primary analytical approach to assess the association between PD and 14 types of cancer (Slob and Burgess, 2020). Using this method, effect estimates for each SNP on exposure and outcome risks were obtained through Wald estimation. Although this method can provide precise estimates, it is susceptible to the influence of invalid IVs and potential pleiotropic effects. Therefore, multiple sensitivity analyses were performed to assess the stability of the association. Firstly, we applied the weighted median (WM) method to ascertain the association, operating under the assumption that a minimum of 50% of the weight originates from valid instruments (Bowden et al., 2016). Secondly, Cochran’s Q test was utilized to assess heterogeneity among estimates from different genetic variants, with a *p*-value below 0.05 indicating significant heterogeneity (Greco et al., 2015). Thirdly, MR-Egger regression was applied to detect the presence of directional pleiotropy by testing whether the intercept statistically differs from zero (Bowden et al., 2015). Fourthly, we utilized the MR Pleiotropy Residual Sum and Outlier (MR-PRESSO) test to detect potential outliers and correction outcomes were obtained by removing outliers (Verbanck et al., 2018). Finally, a leave-one-out analysis was performed to evaluate how the exclusion of individual selected SNPs would affect the aggregate findings (Burgess et al., 2017). In addition, we explored the relationship between subcategories of skin cancer and PD.

MR correlation analysis was adjusted for multiple testing using the Benjamini-Hochberg method to compute corrected false discovery rate (FDR)-adjusted *p*-values (Benjamini and Hochberg, 1995). Additionally, the text mentions unadjusted raw p-values. The significance level for FDR-corrected p-values was set at 0.05. Both the raw *p*-value and the FDR-adjusted p-value were less than 0.05, suggesting a significant correlation; When raw p-values were below 0.05, but the FDR-adjusted p-values exceeded 0.05, these results were deemed to offer suggestive evidence of the reported association. All of the statistical analyses mentioned were conducted in a Windows environment utilizing the “TwoSampleMR” package (version 0.5.8), the “MR-PRESSO” package, and the “forestploter” package in R version 4.3.2.

## 3 Results

**Figure 2:**
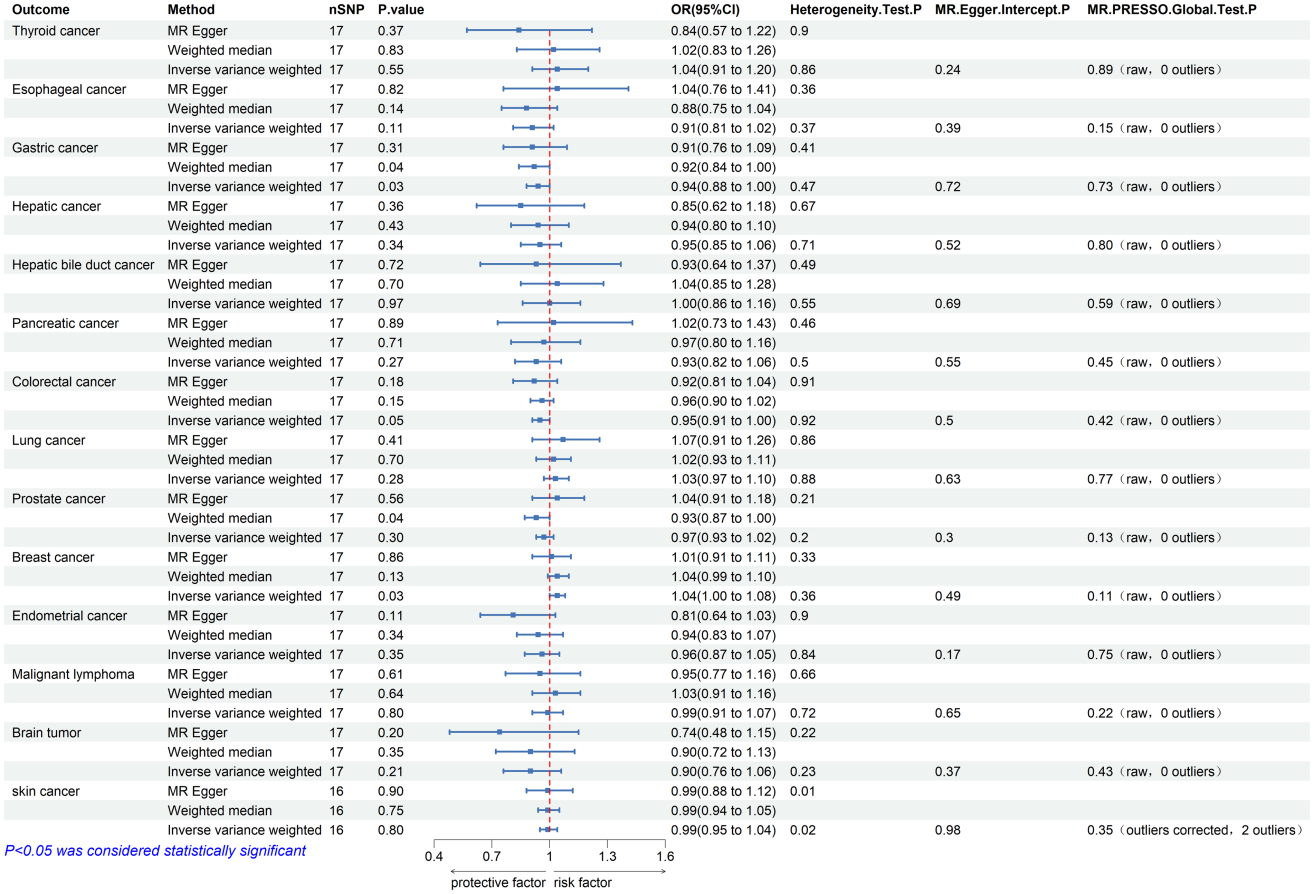
Mendelian randomization assessments of the association between Parkinson’s disease and 14 types of cancer. nSNP, number of single-nucleotide polymorphisms incorporated in the analysis; OR, odds ratio; CI, confidence interval.

**Figure 3:**
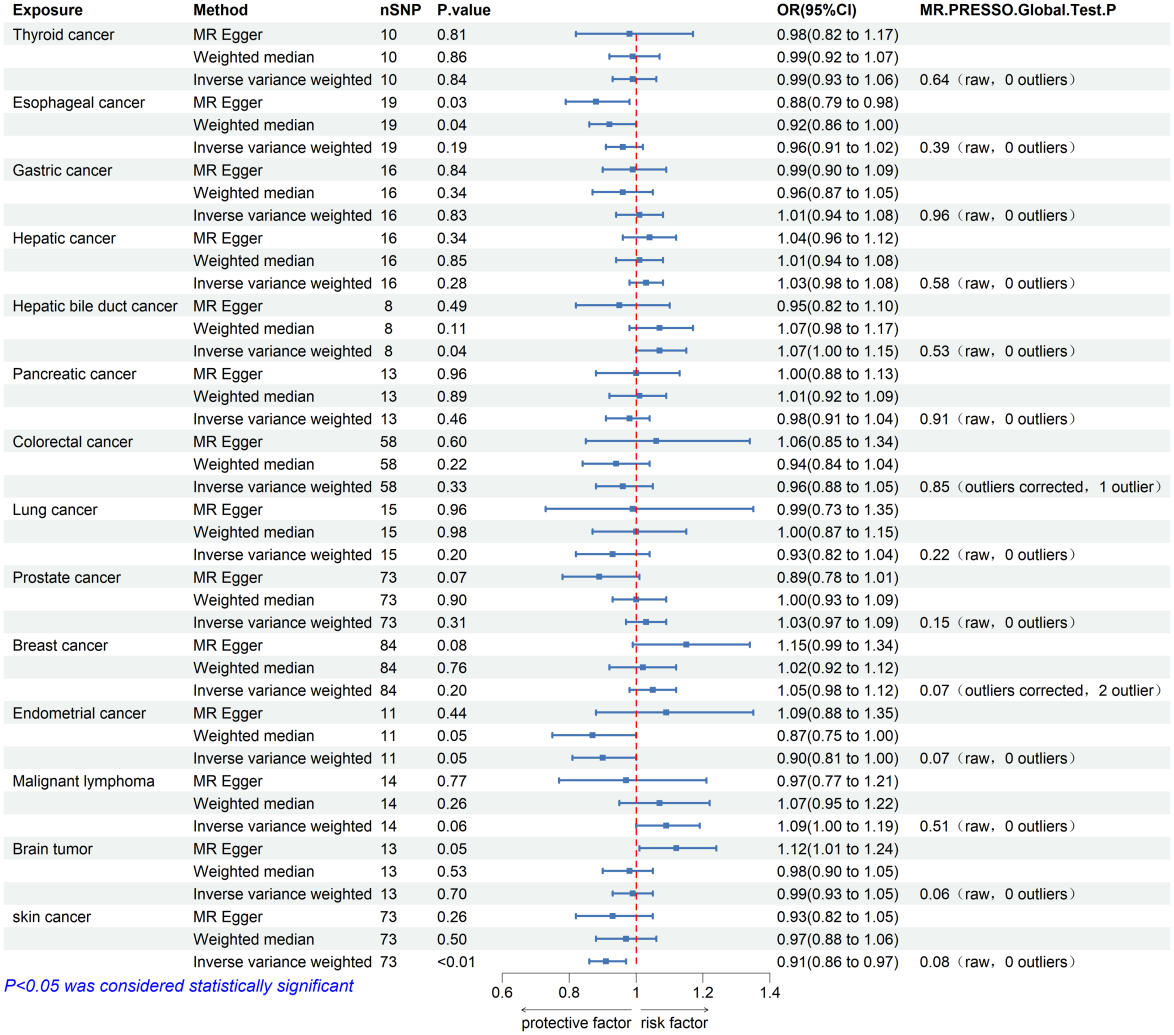
Mendelian randomization assessments of the association between 14 types of cancer and Parkinson’s disease. nSNP, number of single-nucleotide polymorphisms incorporated in the analysis; OR, odds ratio; CI, confidence interval.

Bidirectional TSMR analyses were conducted to assess the association between PD and 14 types of cancer. The number of eligible SNPs associated with the exposure (PD or the 14 types of cancer) is shown in Figures 2, 3, respectively.

### 3.1 The causal effect of Parkinson’s disease on cancer

Initially, 23 SNPs related to PD (22 SNPs when the outcome is skin cancer) were selected from the International Parkinson’s Disease Genomics Consortium (IPDGC) as effective IVs. Following stringent screening, 17 SNPs associated with PD (16 SNPs when the outcome was skin cancer) were identified as effective IVs for analyzing their causal relationships with 14 types of cancer. Detailed data are provided in Table 2. Causal relationship analysis between PD and cancer was conducted using the IVW, Weighted Median (WM), and MR-Egger methods. Statistically significant findings from the IVW model revealed an association between PD and the risk of gastric cancer (odds ratio [OR] = 0.936; 95% confidence interval [CI] = 0.881–0.995; *p* = 0.034; P FDR = 0.239), colorectal cancer (OR = 0.955; 95% CI = 0.912–0.999; *p* = 0.046; P FDR = 0.215), and breast cancer (OR = 1.043; 95% CI = 1.004–1.084; *p* = 0.029; P FDR = 0.402; Figure 2). Since the FDR-adjusted *p*-values for all comparisons exceeded 0.05, this indicates a suggestive association between PD and these three cancers.

**Table 2:** Primary outcomes of MR analysis.

Outcome	Method	nSNPs	OR (95% CI)	*p* value	P FDR
Thyroid cancer	IVW	17	1.044(0.905–1.204)	0.554	0.705
Esophageal cancer	IVW	17	0.911(0.813–1.201)	0.109	0.380
Gastric cancer	IVW	17	0.936(0.881–0.995)	**0.034**	0.239
Hepatic cancer	IVW	17	0.947(0.847–1.058)	0.335	0.521
Hepatic bile duct cancer	IVW	17	1.003(0.864–1.163)	0.972	0.972
Pancreatic cancer	IVW	17	0.931(0.818–1.059)	0.274	0.640
Colorectal cancer	IVW	17	0.955(0.912–0.999)	**0.046**	0.215
Lung cancer	IVW	17	1.034(0.974–1.098)	0.276	0.552
Prostate cancer	IVW	17	0.974(0.927–1.024)	0.303	0.530
Breast cancer	IVW	17	1.043(1.004–1.084)	**0.029**	0.402
Endometrial cancer	IVW	17	0.957(0.873–1.049)	0.348	0.487
Malignant lymphoma	IVW	17	0.989(0.911–1.074)	0.799	0.861
Brain tumor	IVW	17	0.900(0.762–1.063)	0.215	0.601
Skin cancer	IVW	16	0.994(0.948–1.042)	0.796	0.928

Bold values indicated suggestive statistical significance (*p* < 0.05). IVW, inverse variance weighted; nSNPs, number of single-nucleotide polymorphisms incorporated in the analysis; OR, odds ratio; CI, confidence interval; FDR, false discovery rate.

In the sensitivity analysis, the OR values calculated by MR-Egger regression and weighted median methods were consistently aligned in direction with the results of the IVW method. Both Cochran’s Q test and MR-Egger intercept test indicate the absence of heterogeneity (*p* > 0.05) or pleiotropy (P intercept >0.05) between PD and gastric, colorectal, and breast cancer. Furthermore, the MR-PRESSO global test did not indicate substantial pleiotropy (P Global test >0.05). Scatter plots and forest plots visually depict their relationships. Additionally, leave-one-out analysis suggests that the causal relationships between PD and the three cancers are not influenced by any single SNP. The Supplementary materials include leave-one-out analysis, scatter, and forest plots, which are designated as Supplementary Figures 1–3.

### 3.2 The causal effect of cancer on Parkinson’s disease

The causal effect analysis of 14 types of cancer on PD was conducted random-effects IVW, WM, and MR-Egger methods, as shown in Table 3. The findings from the Inverse IVW method suggest that there is no causal link between gastric cancer, colorectal cancer, breast cancer, and PD (*p* > 0.05). However, a suggestive causal relationship is observed between skin cancer and PD (OR = 0.913; 95% CI = 0.857–0.973; *p* = 0.005; P FDR = 0.066; Figure 3). Additionally, due to the inconsistent directions of the three MR methods, the causal relationship between hepatobiliary cancer and PD cannot be reliably determined.

**Table 3:** Primary outcomes of the inverse MR analysis.

Exposure	Method	nSNPs	OR (95% CI)	*p* value	P FDR
Thyroid cancer	IVW	10	0.994(0.935–1.056)	0.840	0.840
Esophageal cancer	IVW	19	0.963(0.910–1.019)	0.191	0.534
Gastric cancer	IVW	16	1.008(0.940–1.080)	0.832	0.896
Hepatic cancer	IVW	16	1.026(0.979–1.075)	0.281	0.491
Hepatic bile duct cancer	IVW	8	1.073(1.002–1.148)	0.043	0.302
Pancreatic cancer	IVW	13	0.975(0.913–1.042)	0.459	0.585
Colorectal cancer	IVW	58	0.958(0.878–1.045)	0.333	0.467
Lung cancer	IVW	15	0.926(0.825–1.041)	0.197	0.394
Prostate cancer	IVW	73	1.028(0.974–1.085)	0.309	0.481
Breast cancer	IVW	84	1.047(0.976–1.123)	0.196	0.457
Endometrial cancer	IVW	11	0.899(0.807–1.001)	0.051	0.239
Malignant lymphoma	IVW	14	1.087(0.997–1.186)	0.059	0.208
Brain tumor	IVW	13	0.988(0.929–1.051)	0.699	0.816
Skin cancer	IVW	73	0.913(0.857–0.973)	**0.005**	0.066

Bold values indicated suggestive statistical significance (*p* < 0.05). IVW, inverse variance weighted; nSNPs, number of single-nucleotide polymorphisms incorporated in the analysis; OR, odds ratio; CI, confidence interval; FDR, false discovery rate.

In the sensitivity analysis, both Cochran’s Q test and the MR-Egger intercept test indicated an absence of heterogeneity (*p* > 0.05) and pleiotropy (P intercept >0.05) in the relationship between skin cancer and PD. Furthermore, the MR-PRESSO global test showed no significant pleiotropy (P Global test >0.05). Scatter plots and forest plots visually depict these relationships. Additionally, leave-one-out analysis reveals that no SNP impacts the causal link between PD and skin cancer. Supplementary materials include leave-one-out analysis plots, scatter plots, and forest plots, all accessible in Supplementary Figure 4.

### 3.3 Association of skin cancer subcategories with Parkinson’s disease

We explored the association between non-melanoma skin cancer (NMSC), melanoma skin cancer (MSC), and Parkinson’s disease. Using data from the UK Biobank (UKB) and the FinnGen Biobank (FG), we analyzed the data using three methods: MR-Egger, weighted median, and inverse-variance weighting. In the UKB dataset, neither NMSC nor MSC showed a significant association with PD (OR = 0.9998, 95% CI = 0.9971–1.0025, *p* = 0.895; OR = 1.0004, 95% CI = 0.9995–1.0012, *p* = 0.381). However, in the FG dataset, there was a significant negative correlation between NMSC and PD (Supplementary Table 1).

## 4 Discussion

In this research, we applied bidirectional TSMR to evaluate the established GWAS dataset, aiming to determine a causal link between PD and cancer in Europeans. We excluded the significant effects of anomalous variants and horizontal pleiotropy on the study results through a series of sensitivity analyses and robustness tests. Our findings indicate a possible causal connection where genetic predispositions to PD are associated with a lower risk of gastric and colorectal cancers, alongside a higher risk of breast cancer. However, this causal relationship is not supported in the reverse analysis. Additionally, in the reverse analysis, suggestive causal relationships between skin cancer and PD are also observed.

In recent years, controversy has arisen regarding the association between PD and cancer. The study by Abhimanyu et al. indicates that in the U.S. population, cancer, particularly skin cancer, may delay the onset of PD (Mahajan et al., 2020). Most European epidemiological studies support a negative link between the risk of developing PD and cancer (Feng et al., 2015; Fang et al., 2021). Similar findings have been observed in studies involving East Asian populations. A cohort study involving 52,009 PD patients in Korea found that individuals with PD had a reduced overall risk of developing cancer, as well as a decreased risk of most types of cancer (Park et al., 2019). Similarly, another study, utilizing claims data from the Korean National Health Insurance Database, arrived at similar conclusions (Kim et al., 2023).

However, emerging evidence suggests a significant association between certain types of cancer and PD. Sugier and colleagues analyzed GWAS datasets from two PD studies and six cancer investigations to explore the genetic links between PD and various cancers. Their findings indicated significant positive associations between PD and several cancers, including melanoma, prostate cancer, and breast cancer (Sugier et al., 2023). These results align closely with those of a previous nationwide association study conducted in the UK (Ong et al., 2014). Despite efforts by researchers to investigate the causal link between PD and cancer using MR methods, consistent positive outcomes have yet to be achieved (Senkevich et al., 2021; Guo et al., 2023; Wang et al., 2023). In this study, we leveraged cancer GWAS data from a meta-analysis involving the UK Biobank and FinnGen, providing initial evidence of a suggestive causal correlation between PD and certain cancers, in line with the majority of previous epidemiological investigations (Rugbjerg et al., 2012; Xie et al., 2017; Tian et al., 2021).

Despite the evident distinctions between PD and cancer, an increasing body of research indicates a potential link between them. To date, several hypotheses have been proposed to explain the potential mechanisms underlying the correlation between PD and the risk of cancer. 1. Gene Mutations: The potential link between PD and certain cancers could be attributed to specific gene mutations. For instance, mutations in genes such as *SNCA* (*α*-synuclein), *ATM* (ataxia telangiectasia mutated), *PARK2* (parkin), *PARK8* (LRRK2), *MC1R* (Melanocortin 1 receptor) and *PTEN* (Phosphatase and tensin homolog) have been identified in both PD and certain cancers. These genes are involved in essential biological processes, including cell cycle control, protein degradation, mitochondrial function, and cellular stress response (Feng et al., 2015; Brundin and Wyse, 2018; Liu et al., 2018; Filippou and Outeiro, 2021; Morris et al., 2024). 2. Chronic inflammation: Persistent inflammatory processes are critical in the development of PD, potentially exacerbating neurodegenerative changes by activating microglial cells and releasing pro-inflammatory factors (Russo et al., 2014; Subhramanyam et al., 2019). Similarly, inflammation and immune responses contribute to the development and metastasis of cancer (Geraldo et al., 2021; Evans et al., 2023). 3. PI3K-AKT–mTOR signaling pathway: This signaling pathway is central to cell growth, proliferation, and survival processes. In PD, abnormal activation of this pathway may influence neuronal survival (Elstner et al., 2011), whereas in cancer, excessive activation of this pathway is a critical factor in tumor growth and invasion (Deng et al., 2023; Glaviano et al., 2023; Duchatel et al., 2024). Investigating these associated mechanisms not only enhances understanding of the complex pathological processes underlying PD and cancer but also holds promise for the development of novel therapeutic strategies. Furthermore, PD is also considered a heterogeneous syndrome, including different clinical and pathological subtypes (Marsili and Mahajan, 2022). This heterogeneity may have a significant impact on research into the association between PD and cancer. Certain subtypes of PD may have a stronger association with specific types of cancer. Some studies have indicated that PD patients with LRRK2 gene mutations have a higher risk of developing colorectal cancer (Wang et al., 2024), which contradicts research that does not take PD subtypes into account (Fang et al., 2021). Therefore, in future studies, identifying and investigating the different subtypes of PD and their associations with specific types of cancer may help to better understand the complex pathophysiological relationship between PD and cancer. This subtype-specific analysis will provide strong support for precision medicine, thereby aiding in the development of more personalized treatment strategies for different patients.

Our MR study possesses several strengths. Firstly, to the best of our knowledge, this MR study represents the first validation of a causal relationship between PD and cancer. In comparison to previous observational studies, MR analysis serves as an effective tool for mitigating potential biases, such as confounding factors and reverse causation, thereby improving the reliability of causal inference. Secondly, it is noteworthy that the GWAS datasets for PD and cancer predominantly involve populations of European ancestry, which helps to minimize the impact of population stratification effects. Thirdly, we utilized diverse estimation models and conducted rigorous sensitivity analyses to ensure the reliability and robustness of the results obtained.

However, we acknowledge several limitations in our study. First, the initial analyses did not produce significant results, indicating the necessity for larger sample sizes of data and more advanced methods to validate our findings. Secondly, our study was conducted on a single population, and further validation is required to generalize the results across diverse ethnicities. Thirdly, despite implementing rigorous steps to identify outlier variants and mitigate horizontal pleiotropy, we cannot entirely eliminate its influence. This may be attributed to the intricate and unclear biological functions of numerous genetic variants.

## 5 Conclusion

The current MR study provides moderate evidence regarding the unidirectional causal relationship between PD and gastric cancer, colorectal cancer, breast cancer, and skin cancer. Furthermore, to further validate and refine these findings, future research necessitates higher-quality GWAS data and the utilization of more advanced methodologies. These enhancements are essential to bolster the study’s statistical power and enhance the precision of the conclusions.

## Data Availability

Publicly available datasets were analyzed in this study. This data can be found at: https://gwas.mrcieu.ac.uk/.
