# Supplementary material for: Causal association between Parkinson’s disease and cancer: a bidirectional Mendelian randomization study
Source: Front Aging Neurosci. 2024 Nov 5;16:1432373. doi: 10.3389/fnagi.2024.1432373 (PMC11573767; doi:10.3389/fnagi.2024.1432373)
Supplement: Supplementary file 3 [file Supplementary_file_3.docx]

Supplementary Material

**(A) (B) (C)**

| 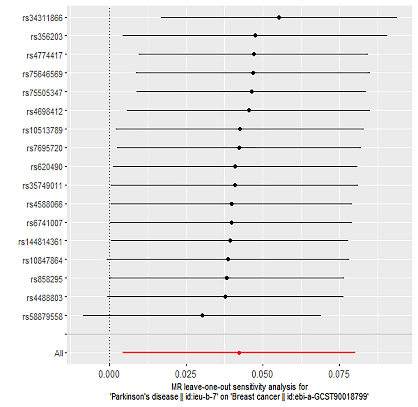 | 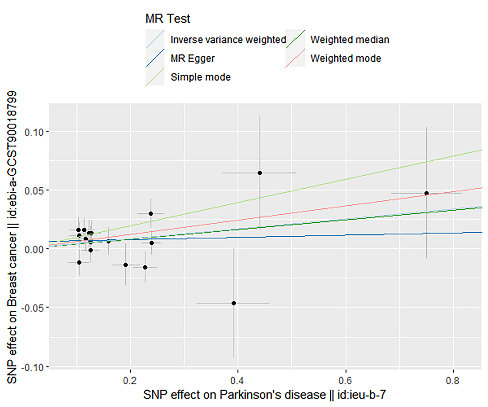 | 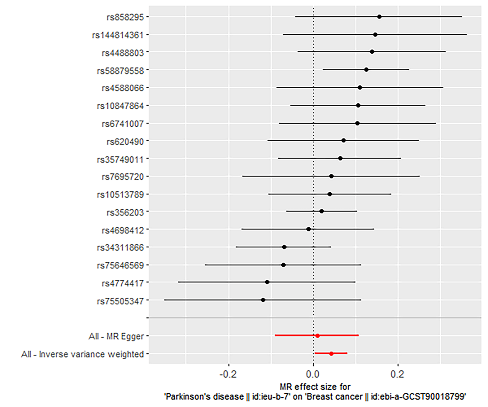 |
| --- | --- | --- |

**Supplementary Figure 3.** The visualization results of MR analysis with Parkinson's disease as the exposure and Breast cancer as the outcome. **(A)**: leave-one-out plots, **(B)**: scatter plots, **(C)**: forest plots.
