# Supplementary material for: Causal association between Parkinson’s disease and cancer: a bidirectional Mendelian randomization study
Source: Front Aging Neurosci. 2024 Nov 5;16:1432373. doi: 10.3389/fnagi.2024.1432373 (PMC11573767; doi:10.3389/fnagi.2024.1432373)
Supplement: Supplementary file 5 [file Table_1.docx]

**Supplementary Table 1 Association of skin cancer subcategories with Parkinson's disease**

| **Outcome** | **Method** | **nSNP** | **P.value** | **or** | **or_lci95** | **or_uci95** |
| --- | --- | --- | --- | --- | --- | --- |
| **NMSC（UKB）** | MR Egger | 20 | 0.6199 | 0.9984 | 0.9920 | 1.0048 |
|  | Weighted median | 20 | 0.6222 | 1.0008 | 0.9977 | 1.0039 |
|  | Inverse variance weighted | 20 | 0.8952 | 0.9998 | 0.9971 | 1.0025 |
| **MSC（UKB）** | MR Egger | 20 | 0.5247 | 1.0007 | 0.9986 | 1.0027 |
|  | Weighted median | 20 | 0.1890 | 1.0008 | 0.9996 | 1.0019 |
|  | Inverse variance weighted | 20 | 0.3810 | 1.0004 | 0.9995 | 1.0012 |
| **NMSC（FG）** | MR Egger | 18 | 0.9152 | 1.0098 | 0.8465 | 1.2046 |
|  | Weighted median | 18 | 0.1683 | 0.9416 | 0.8644 | 1.0258 |
|  | Inverse variance weighted | 18 | **0.0498** | 0.9415 | 0.8865 | 1.0000 |

NMSC: Non-melanoma skin cancer; MSC: Melanoma skin cancer; UKB: UK Biobank; FG: FinnGen Biobank
